# Draft Genome Sequences of Four Escherichia albertii Isolates from Hunted Wild Boars in Switzerland

**DOI:** 10.1128/mra.00135-23

**Published:** 2023-04-12

**Authors:** Michael Biggel, Karen Barmettler, Andrea Treier, Francis Muchaamba, Roger Stephan

**Affiliations:** a Institute for Food Safety and Hygiene, Vetsuisse Faculty, University of Zürich, Zürich, Switzerland; Queens College Department of Biology

## Abstract

Here, we report the genome sequences of four Escherichia albertii isolates that were recovered from hunted wild boars in Switzerland in 2022 and 2023. The genome sizes of KBWS15i, KBWS35i, KBWS50i, and KBSW171i were 4.4 Mbp, 4.5 Mbp, 4.5 Mbp, and 4.7 Mbp, respectively.

## ANNOUNCEMENT

Escherichia albertii is a Gram-negative, nonmotile, non-spore-forming, facultative anaerobic bacterium of the family *Enterobacteriaceae* (1). This zoonotic pathogen has sporadically been associated with gastroenteritis in humans (2, 3), and the wild animal reservoirs are not yet well defined (1). In this study, we screened 52 hunted wild boars from different regions in Switzerland for potential carriage of E. albertii. Fecal samples were collected by the hunters between December 2022 and February 2023 and incubated overnight at 42°C in *Enterobacteriaceae* enrichment (EE) broth (Becton Dickinson). Cultures were then streaked on sheep blood agar (Difco Columbia blood agar base EH; Becton Dickinson) and again incubated overnight at 42°C. Colonies were washed off with 2 mL NaCl (0.85%), and 100 μL thereof was combined with 200 μL Gram-negative lysis buffer. Samples were heated for 50 min at 60°C and then for 10 min at 99°C and centrifuged at 11,000 rpm for 2 min. Supernatants were tested for the presence of the E. albertii-specific cytolethal distending toxin (*Eacdt*) gene by PCR (2). For *Eacdt*-positive samples, a loopful of the washed-off bacterial suspension was streaked on xylose-MacConkey agar plates (3) and incubated overnight at 42°C. Five samples (9.6%) were PCR positive for *Eacdt*; for four of those, E. albertii isolates could be recovered. Matrix-assisted laser desorption ionization–time of flight mass spectrometry (MALDI-TOF MS) (Bruker Daltronics) was used for preliminary genus identification. Genomic DNA was extracted from subcultures obtained from single colonies that had been grown for 24 h at 37°C on sheep blood agar, using the DNeasy blood and tissue kit (Qiagen). Libraries were prepared using the Nextera DNA Flex library preparation kit (Illumina) and sequenced on the Illumina MiniSeq platform (2 × 150 bp). Fastp v0.23.2 (4) was used for read trimming and read quality assessment. Draft assemblies were generated using SPAdes v3.14.1 (5) implemented in Shovill v1.1.0 (https://github.com/tseemann/shovill). The genomes were annotated using the NCBI Prokaryotic Genome Annotation Pipeline (PGAP) v6.4 (6). Species analysis and multilocus sequence typing (MLST) were performed using rMLST (7) and MLST v2.22.0 (https://github.com/tseemann/mlst) (Escherichia coli Achtman scheme), respectively. AMRFinder v3.10.24 was used for the detection of resistance genes (8), and ABRicate v1.0.1 (https://github.com/tseemann/abricate) (minimum coverage, 50%; identity, 70%) was used in combination with Virulence Factor Database (VFDB) set B (9) for the detection of virulence genes. Default parameters were used for all software unless otherwise stated.

The draft assembly sizes of KBWS15i, KBWS35i, KBWS50i, and KBSW171i ranged between 4.4 and 4.7 Mbp. Detailed assembly and sequencing metrics are given in Table 1. The four isolates were typed as E. albertii sequence type 14288 (ST14288), ST4619, ST14289, and ST4170, respectively. Whereas ST14288 and ST14289 were newly assigned, multiple isolates among ST4619 entries in EnteroBase (10) originate from Sus scrofa, indicating a host association. None of the isolates harbored acquired antimicrobial resistance genes, the aerobactin biosynthetic gene cluster (*iuc*), or Shiga toxin-encoding genes, which are occasionally found in E. albertii. This is the first study showing that wild boars are a reservoir for E. albertii.

**TABLE 1:** Sequencing data and assembly metrics

Isolate	Total no. of reads	Total read length (Mbp)	Mean coverage (×)	Genome size (bp)	No. of contigs	*N*_50_ (bp)	GC content (%)	Genome assembly accession no.	SRA accession no.
KBWS15i	1,359,691	201.0	45	4,426,885	37	289,012	49.81	GCA_028807055.1	SRR23495216
KBWS35i	1,526,165	228.3	50.1	4,515,700	57	360,268	49.7	GCA_028807295.1	SRR23495215
KBWS50i	1,318,255	196.5	43.2	4,503,808	77	311,576	49.85	GCA_028806815.1	SRR23495214
KBSW171i	1,828,605	272.7	57.2	4,724,403	63	353,379	49.69	GCA_028807025.1	SRR23495217

This study was performed in accordance with the Swiss Animal Welfare Act (SR 455). No animal was killed for this study. The fecal samples were collected after routine hunting of wild boars.

### Data availability.

Assemblies and read data were deposited in the NCBI database under BioProject accession number PRJNA879956. Individual accession numbers are listed in Table 1.
